# The prevalence of muscular dystrophy and spinal muscular atrophy in Croatia: data from national and non-governmental organization registries

**DOI:** 10.3325/cmj.2019.60.488

**Published:** 2019-12

**Authors:** Željka Draušnik, Ivan Cerovečki, Vesna Štefančić, Sandra Mihel, Ranko Stevanović, Nina Barišić, Hana Matković, Matea Melša, Marica Mirić, Neda Pjevač, Tomislav Benjak

**Affiliations:** 1 Division of Public Health, Croatian Institute of Public Health, Zagreb, Croatia; 2 Department of Social Medicine and Epidemiology, University of Rijeka, Rijeka, Croatia; 3 Department of Pediatrics, University Hospital Centre Zagreb, Zagreb, Croatia; 4 Health Center Zagreb “Center,” Zagreb, Croatia; 5 Croatian Muscular Dystrophy Association, Zagreb, Croatia; 6 Department of Educational Technology and Educational Multimedia Center, “Andrija Štampar” School of Public Health, University of Zagreb School of Medicine, Zagreb, Croatia

## Abstract

**Aim:**

To determine the prevalence of muscular dystrophy (MD) and spinal muscular atrophy (SMA) in Croatia by use of multiple epidemiological tools.

**Methods:**

This epidemiological study collected data from three national patient registries and one database of a non-governmental organization (NGO) of MD and SMA patients. The study involved all individuals who either had undergone hospital treatment for MD or SMA, had consulted their primary health care providers for MD- and SMA-related symptoms, were listed as disabled due to MD or SMA, or were members of the mentioned NGO in 2016. In order to prevent double entries, we created a new database of all living individuals, each with a unique identification number. The prevalence rates for 2016 were calculated by age and sex groups.

**Results:**

There were 926 patients diagnosed with MD (544 men). Most men diagnosed with MD were in the age group 10-19, whereas most women were in the age group 50-59. MD prevalence in Croatia was 22.2 per 100 000 population. There were 392 patients diagnosed with SMA (198 men). Most men with SMA were in the age group 50-59, whereas most women were in the age group 60-69. SMA prevalence in Croatia was 9.3 per 100 000 population.

**Conclusion:**

SMA prevalence rate in Croatia is similar to SMA prevalence worldwide. However, MD prevalence rate is higher than worldwide estimates. This difference could be attributed to the fact that we could not confirm whether every patient registered in these databases actually met the diagnostic criteria for MD and SMA.

Muscular dystrophies (MD) are rare disorders, affecting fewer than 5 per 10 000 population (1). The overall worldwide prevalence of all types of MD was reported to be 16.1 per 100 000 population (1 in 6200 population) (2). The combined prevalence of all types of MD in the United Kingdom was between 19.8 and 25.1 per 100 000 person-years (3). However, the prevalence of specific MD types varies significantly.

Spinal muscular atrophy (SMA) shares a number of common symptoms with MD but has a different etiology. The estimated incidence of SMA worldwide is 1 in 10 000 live births (4). Both MD and SMA are diagnosed based on clinical features and increased creatine kinase levels and subsequently confirmed by genetic testing and/or muscle biopsy. MDs and SMA remain incurable diseases with high unmet medical needs.

Country- and age-specific disease prevalence is usually determined based on data from national and international patient registries and care and trial site registries. When national registries are not complete or available, there are other possibilities to determine epidemiologic indicators pertaining to a particular disease. Data on health care indicators in Croatia are aggregated and statistically analyzed in the Croatian Institute of Public Health (CIPH) (5,6), which also registers individual disease cases in various patient registries and morbidity databases. Unambiguous linking of data contained in all CIPH registries and databases has recently been enabled by advances in anonymization techniques. Moreover, CIPH has been granted access to data in the Central Health Information System of Croatia (CEZIH) database, which collects all information on primary health care provider services. This has enabled researchers to more accurately determine the number of MD and SMA patients, rather than to determine only the pattern of health care services they use.

There has been some research on the prevalence of specific MD in Croatia (7-9), but no data on the total prevalence of MD in Croatia has been published. In addition, previous studies conducted in Croatia involved only smaller regional subpopulations. When it comes to research on SMA, no studies have so far been conducted in Croatia. The aim of this study was to determine the number of patients diagnosed with MD and SMA in Croatia based on data contained in available registries/databases. This should facilitate resource allocation in the health care system and provision of appropriate and timely medical care to all patients with MD and SMA.

## Methods

The following data sources were used:

1) A database of all patients who underwent hospital treatment in Croatia in 2016 (In-patient Statistics Form database, BSO),

2) A database of all medical services provided by primary health care providers in Croatia in 2016 (Central Health Information System of Croatia, CEZIH),

3) The Disabled Persons Registry (ROI) of the CIPH, and

4) A database of all members of the Croatian Muscular Dystrophy Association (SDDH).

All of the listed sources contain information on patients’ age, sex, and residence, as well as their personal identification number (OIB), which serves as a unique identifier. Additionally, the CEZIH database includes the International Statistical Classification of Diseases and Related Health Problems (ICD) code attributed to the patient and information about prescribed drugs and referrals, whereas the BSO database contains data on the duration of hospital treatment and the associated ICD code. The ROI comprises information about the disability extent and cause (as coded in ICD). The SDDH database contains no additional information except the demographic data and OIB identifier; in order to acquire SDDH membership, applicants have to provide a physician’s certificate proving the diagnosis of MD or SMA.

Data were analyzed during September 2018 in the Division for Public Health of the CIPH. The study involved all individuals who had undergone hospital treatment for MD or SMA (BSO database), had consulted their primary health care providers for MD-related or SMA-related symptoms (CEZIH database), were listed as disabled due to MD or SMA in the ROI, or were SDDH members. The inclusion criterion was the diagnosis of MD or SMA as per the International Statistical Classification of Diseases and Related Health Problems (ICD) codes G71 and G12, respectively. However, we were not able to confirm whether every person with ICD codes G71 or G12 met the diagnostic criteria for MD or SMA, respectively. Deceased patients and patients with residence outside of Croatia (as verified in the national health insurance registry) were excluded.

The data were extracted from BSO, CEZIH, ROI, and SDDH databases with SQL Server Management Studio 2012 software (Microsoft, Redmond, WA, USA) and analyzed with Microsoft Excel 2010. To prevent double entries, all individuals were identified using their OIB number. Similar methods of quadrangulating data sources have been used in other studies (10,11). This study was approved by the Ethics Committee of the CIPH on February 1, 2018, under the Registration number 381-10-18-2.

## Results

There were 926 MD patients (544 or 58.7% men, sex ratio: 1.42) (Table 1). Most men diagnosed with MD (95 individuals) were in the age group 10-19, whereas most women (75 individuals) were in the age group 50-59. The overall MD prevalence in Croatia was 22.2 patients per 100 000 population. The distribution of prevalence rates by age groups was roughly bimodal, with the greatest prevalence rates in the age groups 10-19 and 40-49 in both sexes. The youngest MD patient was a male patient aged 2 years, whereas the oldest was a female patient aged 91.

**Table 1:** Patients diagnosed with muscular dystrophy by sex and age groups*^†^

Age group (years)	Male population		Female population		General population	
	patients	PR	patients	PR	patients	PR
0-9	44	21.1	23	11.7	67	16.5
10-19	95	42.8	41	19.5	136	31.4
20-29	83	32.6	40	16.3	123	24.6
30-39	72	24.8	40	14.2	112	19.6
40-49	70	25.1	58	20.9	128	23.1
50-59	82	27.8	75	24.3	157	26.0
60-69	60	23.4	68	23.4	128	23.4
70 +	38	18.1	37	10.6	75	13.4
**Total**	**544**	**27.0**	**382**	**17.7**	**926**	**22.2**

*PR – prevalence rate per 100 000 population. †Data on the estimated number of inhabitants in particular sex and age groups (mid-2016 estimates, published on July 27, 2017) retrieved from the web site of the Croatian Bureau of Statistics (*https://www.dzs.hr/Hrv_Eng/Pokazatelji/Procjene%20stanovnistva.xlsx*; accessed on August 27, 2018).

The ROI included data on 62.6% of MD patients and SDDH database on 34.9% of MD patients. Other data sources comprised data on less than 10% of patients (Table 2). Moreover, the ROI included data on 518 patients not listed in any other data source (55.9% of the total number of MD patients). SDDH database included data on 323 patients not listed in other databases (34.9%); the proportions in other databases were minor (Table 3).

**Table 2:** The number of muscular dystrophy patients in individual data sources

Data source	No. (%) of muscular dystrophy patients in the respective database (N = 926)	No. (%) of muscular dystrophy patients only in the respective database (N = 926)
**Disabled Persons Registry**	580 (62.6)	518 (55.9)
**In-patient Statistics Form database**	12 (1.3)	0 (0.0)
**Central Health Information System of Croatia**	65 (7.0)	22 (2.4)
**Croatian Muscular Dystrophy Association**	345 (37.3)	323 (34.9)

**Table 3:** The number of muscular dystrophy patients in multiple data sources

Number of databases	Number (%) of muscular dystrophy patients in the databases (N = 926)
Two databases*	
ROI + BSO	5 (0.5)
ROI + CEZIH	31 (3.3)
BSO + CEZIH	1 (0.1)
BSO + SDDH	0 (0.0)
CEZIH + SDDH	0 (0.0)
ROI + SDDH	14 (1.5)
**Three databases**	
ROI + BSO + CEZIH	4 (0.4)
ROI + BSO + SDDH	1 (0.1)
BSO + CEZIH + SDDH	0 (0.0)
ROI + CEZIH + SDDH	6 (0.6)
**Four databases**	
ROI + BSO + CEZIH + SDDH	1 (0.1)

*ROI – Disabled Persons Registry; BSO – In-patient Statistics Form database; CEZIH – Central Health Information System of Croatia; SDDH – Croatian Muscular Dystrophy Association.

There were 389 SMA patients (97 or 50.6% men, sex ratio: 1.03) (Table 4). Most men diagnosed with SMA (46 individuals) were in the age group 50-59, whereas most women (47 individuals) were in the age group 60-69. The overall SMA prevalence in Croatia was 9.3 patients per 100 000 population. The greatest prevalence rates were observed in patients older than 50 years. The youngest SMA patients (two boys and one girl) were younger than one year, whereas the oldest patient was a female patient aged 96.

**Table 4:** Patients diagnosed with spinal muscular atrophy by sex and age groups*^†^

Age group (years)	Male population		Female population		General population	
	patients	PR	patients	PR	patients	PR
0-9	8	3.8	8	4.1	16	3.9
10-19	14	6.3	17	8.1	31	7.2
20-29	17	6.7	14	5.7	31	6.2
30-39	21	7.2	14	5.0	35	6.1
40-49	19	6.8	31	11.2	50	9.0
50-59	46	15.6	19	6.2	65	10.8
60-69	25	9.7	47	16.1	72	13.1
70 +	47	22.4	42	12.0	88	15.8
**Total**	**197**	**9.8**	**192**	**8.9**	**389**	**9.3**

*PR – prevalence rate per 100 000 population.

†Data on the estimated number of inhabitants in particular sex and age groups (mid-2016 estimates, published on July 27, 2017) retrieved from the web site of the Croatian Bureau of Statistics (*https://www.dzs.hr/Hrv_Eng/Pokazatelji/Procjene%20stanovnistva.xlsx*; accessed on August 27, 2018).

The CEZIH database included data on by far the greatest proportion of SMA patients, accounting for 92.8% of the total number of SMA patients. The SDDH database comprised data on 23.7%, the BSO database on 7.5%, and the ROI database on 74.8% of SMA patients (Table 5). The CEZIH database also included data on 236 patients not listed in any of the other data sources (60.7% of the total number of SMA patients), whereas other databases accounted for a small proportion of SMA patients (Table 6).

**Table 5:** The number of spinal muscular atrophy patients in individual data sources

Data source	No. (%) of spinal muscular atrophy patients in the respective database (N = 389)	No. (%) of spinal muscular atrophy patients only in the respective database (N = 389)
**Disabled Persons Registry**	291 (74.8)	27 (6.9)
**In-patient Statistics Form database**	29 (7.5)	8 (2.1)
**Central Health Information System of Croatia**	361 (92.8)	236 (60.7)
**Croatian Muscular Dystrophy Association**	92 (23.7)	20 (5.1)

**Table 6:** The number of spinal muscular atrophy patients in multiple data sources

Number of databases	No. (%) of spinal muscular atrophy patients in the databases (N = 389)
**Two databases**	
ROI + BSO	2 (0.5)
ROI + CEZIH	0 (0.0)
BSO + CEZIH	7 (1.8)
BSO + SDDH	9 (2.3)
CEZIH + SDDH	58 (14.9)
ROI + SDDH	5 (1.3)
**Three databases**	
ROI + BSO + CEZIH	1 (0.3)
ROI + BSO + SDDH	2 (0.5)
BSO + CEZIH + SDDH	14 (3.6)
ROI + CEZIH + SDDH	0 (0.0)
**Four databases**	
ROI + BSO + CEZIH + SDDH	0 (0.0)

*ROI – Disabled Persons Registry; BSO – In-patient Statistics Form database; CEZIH – Central Health Information System of Croatia; SDDH – Croatian Muscular Dystrophy Association.

## Discussion

The MD prevalence rate in Croatia is higher than the worldwide estimates, but similar to the prevalence obtained in a systematic review from the United Kingdom (19.8-25.1/100 000 population). Such a high prevalence rate, other than by inherited factors, could be explained by the fact that the ICD-10 codes in the databases are not confirmed by a review of medical records. In a number of cases, MD or SMA may have only been a provisional or working diagnosis, which was not confirmed or revised after further examinations or laboratory tests. This explains the discrepancies in the prevalence between different registries or databases. Such discrepancies stress the need for regular clinical audits, both in diagnostic and in therapeutic medical specialties, with an aim to improve patients’ outcomes, overall efficiency, and cost-effectiveness. Nevertheless, regardless of whether the diagnosis will eventually be confirmed, if we want to efficiently manage the resources, we need to know how many people use the health care system under the diagnosis of MD or SMA.

The mean annual direct cost of MD per patient in Germany, Italy, United Kingdom, and United States, is approximately 10, 8, 16, and 7 times higher than the mean per-capita health expenditure in these countries, respectively (12,13). Furthermore, medical needs and health care resource use increase as MD progresses, with a corresponding increase in costs, especially after the age of 14. Higher costs in senior age groups are primarily attributable to a greater number of outpatient visits and office visits (12,13). Advances in supportive care and equipment have improved patients’ survival and quality of life, however, the substantial cost of these services has increased the financial burden on their families and the health care system. Moreover, significant expenses incurred during diagnostic procedures also contribute to the overall cost of patient management in MD (14).

The total cost of health care for SMA patients in the USA and Germany before the introduction of nusinersen therapy was US$ 957 million and € 106.2 million per year, respectively (15). In Spain, the direct non-health care costs of SMA were higher than direct health care costs and were associated with reduced caregiver quality of life (15). This was predominantly related to informal caregiving, indicating that it is important to take into account significant hidden or unmeasured costs. Caring for a child with SMA can entail significant financial costs associated with family caregiving, employment constraints, caregiver burden, social limitations, and reduced caregiver well-being, all of which often remain unrecognized in quantitative studies (15).

Nusinersen therapy, approved in the USA in 2016, is the first efficient pharmacological treatment for SMA (16). Since then, nusinersen has been approved for use in numerous European countries, including Croatia. However, its cost remains highly prohibitive, even in highly developed countries, as it amounts to up to US$ 750 000 for the first year of treatment and US$ 375 000 for each subsequent year (17). A newly approved SMA medication, onasemnogene abeparvovec, is even more costly: applied as one-time treatment, a single dose costs US$ 2.125 million (18). The costs of emerging treatment methods for SMA necessitate that the efficiency of existing treatments is investigated and possible new treatment options are considered.

A limitation of this study is a possible incorrect use of ICD code G71 and G12, designating MD and SMA, respectively, in BSO and CEZIH databases. The database used in this study was formed by aggregating data entries found in multiple databases, rather than by directly examining patient histories. Consequently, it was impossible to confirm whether every patient registered in these databases under the ICD codes G71 or G12 actually met the diagnostic criteria for these entities. Data from the ROI and SDDH databases were confirmed by reviewing medical records, while the data from BSO database only refer to the principal diagnosis, ie, the condition that caused the hospital admission. However, since we used several registries/databases, these discrepancies could not have significantly affected the results.

Although the current SMA prevalence in Croatia is in line with the worldwide estimates (9.3 per 100 000 population vs 10.0 per 100 000 population), the MD prevalence rate is higher (22.2 per 100 000 population vs 16.1 per 100 000 population). Linking anonymized data extracted from multiple databases administered by CIPH or accessible therein allowed us to determine relevant epidemiological indicators. These are necessary for a rational management of limited human and financial resources available in the Croatian health care system. Epidemiological assessments and consequent resource planning could be further improved by establishing a national registry of MD patients.
